# 781 Multimodal Virtual Reality Encouragement

**DOI:** 10.1093/jbcr/irac012.332

**Published:** 2022-03-23

**Authors:** Mini Thomas, Hayley Blacker

**Affiliations:** UCI Health, Orange, California; UCI Health, Irvine, California

## Abstract

**Introduction:**

Burn units’ nurses develop creative methods to mitigate unpleasant experiences. Peer reviewed research has affirmed that non-pharmacological stimuli can distract patients from noxious procedures. Therefore, novel virtual reality was introduced as a nurse-driven modality to improve patient care. Multiple approaches encouraged staff utilization of this exciting, yet unfamiliar, technology.

**Methods:**

A needs assessment concluded that patients experience moderate pain and some anxiety during wound care - indicating an opportunity to explore virtual reality. The virtual reality headset was introduced to nurses via in-services to encourage hands-on experience. The following month, an interactive “VR Superstars” poster was developed as a visual cue to motivate nurses to attempt the new product. Whenever a nurse used the device with a patient, a star sticker was applied next to his/her name. Next, a resource binder was created: This binder included the device protocol, patient inclusion criteria, educational handouts to assist staff with introduction to patients, and surveys. Biweekly emails encouraged virtual reality use and acknowledged staff members who proactively utilized it. The multidisciplinary team also discussed virtual reality at daily rounds.

**Results:**

To assess virtual reality’s effectiveness, patients completed surveys after each use. At the end of the first month, staff implemented virtual reality only three times - as identified by the number of surveys. Upon completion of the multi-modal staff encouragement, virtual reality use doubled. By the third month, nurses implemented the technology on six occasions within the month.

**Conclusions:**

The multi-modal approach ultimately familiarized nurses. These techniques contributed to increasing staff experience, thereby improving staff confidence to utilize a new product. As exposure increased, nurses reported more excitement to introduce the product to patients. Due to the implementation of multiple motivators, nurses are more readily implementing an intervention to benefit a patient’s experience.

